# Risk factors for *Pneumocystis jirovecii* pneumonia (PJP) in kidney transplantation recipients

**DOI:** 10.1038/s41598-017-01818-w

**Published:** 2017-05-08

**Authors:** Su Hwan Lee, Kyu Ha Huh, Dong Jin Joo, Myoung Soo Kim, Soon Il Kim, Juhan Lee, Moo Suk Park, Young Sam Kim, Se Kyu Kim, Joon Chang, Yu Seun Kim, Song Yee Kim

**Affiliations:** 1 Division of Pulmonology, Department of Internal Medicine, Severance Hospital, Institute of Chest Diseases, Yonsei University College of Medicine, Seoul, Republic of Korea; 2 Department of Surgery, Research Institute for Transplantation, Yonsei University College of Medicine, Seoul, Republic of Korea; 3 Division of Pulmonary and Critical Care Medicine, Department of Internal Medicine, Ewha Medical Research Institute, Ewha Womans University School of Medicine, Seoul, Korea

## Abstract

*Pneumocystis jirovecii* pneumonia (PJP) is a potentially life-threatening infection that occurs in immunocompromised patients. The aim of this study was to evaluate risk factors for PJP in kidney transplantation recipients. We conducted a retrospective analysis of patient data from 500 consecutive kidney transplants performed at Severance Hospital between April 2011 and April 2014. Eighteen kidney transplantation recipients (3.6%) were diagnosed with PJP. In the univariate analysis, acute graft rejection, CMV infection, use of medication for diabetes mellitus, and lowest lymphocyte count were associated with PJP. Recipients who experienced acute graft rejection (odds ratio [OR] 11.81, 95% confidence interval [CI] 3.06–45.57, *P* < 0.001) or developed CMV infection (OR 5.42, 95% CI 1.69–17.39, *P* = 0.005) had high odds of PJP in multivariate analysis. In the acute graft rejection subgroup, patients treated with anti-thymocyte globulin (ATG) had significantly higher odds of PJP (OR 5.25, 95% CI 1.01–27.36, *P* = 0.006) than those who were not. Our data suggest that acute graft rejection and CMV infection may be risk factors for PJP in kidney transplant patients. The use of ATG for acute graft rejection may increase the risk of PJP.

## Introduction

*Pneumocystis jirovecii* is an opportunistic fungal pathogen^1^. *Pneumocystis jirovecii* pneumonia (PJP) previously known as *Pneumocystis carinii* pneumonia is a potentially life-threatening infection that occurs in immunocompromised patients^1, 2^. In the absence of prophylaxis, PJP occurs in approximately 5–15% of transplant patients, depending on the transplanted organ or transplant center^2, 3^.

In kidney transplantation, PJP is a very serious risk factor for graft loss and patient mortality^4, 5^. In the absence of appropriate treatment, the mortality rate of PJP is 90–100%, and can be as high as 50% despite adequate therapy^6, 7^. Therefore, several guidelines—such as the Kidney Disease Improving Global Outcomes (KDIGO) guideline, the European Renal Best Practice guideline, and other reports— usually recommend PJP prophylaxis by using TMP/SMX for 3–6 months after renal transplantation^8–10^. The incidence rate of PJP has decreased with the use of prophylaxis, however, an increasing number of PJP outbreaks in kidney transplant centers has been reported worldwide in recent years^4, 5, 11^. The causes of those outbreaks have not fully been evaluated.

Risk factors for the development of PJP in kidney transplant patients are still not confirmed. The overall load of immunosuppressive therapy, higher donor age, higher recipient age, lymphopenia, previous cytomegalovirus (CMV) infection, or treatment used for episodes of graft rejection have been reported as risk factors for PJP in kidney transplant patients^12–17^. However, factors identified in some studies are not always confirmed in other studies. Thus, further research is needed to evaluate the risk factors for PJP in kidney transplant patients in the era of routine PJP prophylaxis. The aim of this study was to evaluate the risk factors for PJP in kidney transplantation recipients.

## Patients and Methods

### Study design and population

This single center, retrospective clinical study included all kidney transplant patients aged ≥18 years who underwent kidney transplantation at the Severance Hospital, a 2000-bed university tertiary referral hospital in South Korea, from April 2011 to April 2014. During this period, 500 patients underwent kidney transplantation; they were followed up until October 2015. We divided the patients into two groups according to the occurrence of PJP—the case group developed PJP whereas the control group did not—and then compared the groups. In our center, all kidney transplant recipients receive PJP prophylaxis using trimethoprim/sulfamethoxazole (TMP/SMX) 160/800 mg *per* day for 12 months post-transplantation. After transplantation, patients received a tacrolimus-based combination regimen or a cyclosporine-based combination regimen for maintaining immunosuppression.

### Data collection

Data from all kidney transplant recipients were collected from the hospital’s electronic medical records. Clinical data on mortality, development of PJP, demographic characteristics, graft origin (deceased *vs*. living), immunosuppressive regimen, data about acute graft rejection such as frequency of such episodes or treatment received, smoking status, history of infections including CMV, BK virus, hepatitis, tuberculosis (TB), and comorbidities were evaluated.

### Definition

We defined PJP when the following two conditions were satisfied; first, a positive result on *Pneumocystis jirovecii* real-time polymerase chain reaction (PCR) testing or direct immunofluorescence testing of microbiological samples (sputum, tracheal aspirate, bronchial washing fluid or bronchoalveolar lavage fluid) and second, identification of lung infiltration on chest computed tomography (CT)^18–20^.

CMV infection was defined as a fourfold elevation of the CMV PCR titer and the use of CMV medication (ganciclovir or valganciclovir). Smoking status was categorized as ever smoker or never smoked; the latter category included those who smoked fewer than 100 cigarettes in their lifetime. Development of acute graft rejection was established by biopsy of the transplanted kidney. Steroid pulse therapy (methylprednisolone [500 mg/day × 4 for 5 days]) was considered the first line therapy for acute graft rejection. When there was an inadequate response to steroid pulse therapy, an anti-thyomcyte globulin (ATG) was used. Lowest lymphocyte count was reviewed during follow-up in the PJP negative group after first hospital discharge and within 1 month before diagnosis of PJP in the PJP group. Clinical characteristics and events were reviewed until the last follow-up date in the PJP negative group and were reviewed until the date of PJP development in the PJP group.

### Ethical approval

The study protocol was approved by the Institutional Review Board (IRB) of Severance Hospital (IRB number: 4-2015-1051). All methods were performed in accordance with the relevant guidelines and regulations. Informed consent was waived by the IRB because of the study’s retrospective nature.

### Statistical analysis

Statistical analysis was performed using SPSS version 20 (IBM, Armonk, New York, USA). Data are described as medians (interquartile range [IQR]) or numbers (*percentages*). The *Chi-squared* and Fisher’s exact test or the Mann-Whitney test was used to assess differences between two groups. The independent risk for PJP was identified using logistic regression modeling. The fundamental variable (such as age, gender, BMI or kidney transplantation type) and variable which were meaningful risk factors in univariate analysis were used in multivariate analysis. A two-tailed *P*-value < 0.05 was considered statistically significant.

## Results

### Characteristics of the overall study population and patients diagnosed with PJP

Demographic and clinical characteristics of the total study population are presented in Table 1. Overall, 500 patients were enrolled over the 3-year study period, with a similar number enrolled each year. The median age of all recipients was 47 years (range, 18–71) and men accounted for 61.4%. The major cause of kidney transplantation was hypertension (39.6%); the other cause was diabetes mellitus (17.0%). Transplanted kidneys were sourced more often from living than deceased donors (64.4% living *vs*. 35.6% deceased). In terms of immunosuppressive medication, 440 (88%) patients received a tacrolimus-based combination regimen and 60 (12%) received a cyclosporine-based combination regimen. The median duration of follow up was 36.2 month (range, 18.4–54.6).

**Table 1:** Demographic and clinical characteristics of total study population and PJP patients.

Characteristics	N = 500
Sex, N (%)	
Men	307 (61.4)
Women	193 (38.6)
Age, years (median, range)	47 (18–71)
Transplantation era, N (%)	
2011.4–2012.3	167 (33.4)
2012.4–2013.3	158 (31.6)
2013.4–2014.4	175 (35.0)
BMI, kg/m2 (median, IQR)	22 (20.1–24.2)
KT type, N (%)	
Deceased	178 (35.6)
Living	322 (64.4)
Re-transplantation, N (%)	41 (8.2)
Primary underlying disease, N (%)	
Polycystic kidney	17 (3.4)
HTN	198 (39.6)
DM	85 (17.0)
IgA nephropathy	63 (12.6)
Autoimmune disease	7 (1.4)
Chronic glomerulonephritis	36 (7.2)
Nephrotic syndrome	41 (8.2)
Recurrent pyelonephritis/other	21 (4.2)
Unknown	32 (6.4)
Immunosuppressive agent, N (%)	
Cyclosporine based regimen	60 (12.0)
Tacrolimus based regimen	440 (88.0)
Follow up duration, month (median, range)	36.2 (18.4–54.5)
Development of PJP, N (%)	18 (3.6)
Interval between PJP and graft, month (median IQR)	17.4 (11.2–27.9)
−12 month, N (%)	4 (22.2)
12–24 month, N (%)	7 (38.9)
24–36 month, N (%)	6 (33.3)
36- month, N (%)	1 (5.6)
Treatment medication of PJP, N (%)	
TMP-SMX alone	13 (72.2)
TMP-SMX prior to primaqiune + clinadamycin	4 (22.2)
TMP-SMX prior to pentamidine	1 (5.6)

Abbreviations: PJP, *Pneumocystis jirovecii* pneumonia; IQR, interquartile range; BMI, body mass index; KT, kidney transplantation; HTN, hypertension; DM, diabetes mellitus; TMP-SMX, trimethoprim/sulfamethoxazole.

Eighteen patients were diagnosed with PJP during the study period, and most cases developed 12–24 months after kidney transplantation. All patients diagnosed with PJP were started on TMP/SMX. Five patients required second-line medication because of disease progression despite TMP-SMX use; four were treated with primaquine and clindamycin, while one was treated with pentamidine. Twelve (66.7%) patients with PJP improved after treatment, with no sequelae. However, three (16.7%) patients had residual sequelae and three (16.7%) died. Table 2 describes clinical characteristics and prognosis of PJP patients.

**Table 2:** Clinical characteristic and prognosis of PJP patients in kidney transplantation.

	Sex	Age	Donor type	Rejection number	CMV infection	Immunosuppression	Treatment	Interval between rejection and PCP	Outcome
Case1	Male	42	Living	1	Yes	Tac, Cor, MMF	TMP-SMX → Pentamidine	3.3 month	Die
Case2	Male	35	Living	4	Yes	Tac, Cor, Miz	TMP-SMX → Primaquine + Clindamycin	3.4 month	Recover
Case3	Male	54	Living	1	No	Tac, Cor, MMF	TMP-SMX	6.9 month	Die
Case4	Male	51	Deceased	0	No	Tac, Cor, MMF	TMP-SMX	—	Recover
Case5	Male	58	Deceased	1	No	Cys, Cor, MMF	TMP-SMX	11 month	Graft fail
Case6	Male	50	Deceased	1	Yes	Tac, Cor, MMF	TMP-SMX	6.2 month	Recover
Case7	Male	53	Deceased	1	No	Tac, Cor, MMF	TMP-SMX	29 month	Graft fail
Case8	Female	55	Living	1	Yes	Tac, Cor, MMF	TMP-SMX → Primaquine + Clindamycin	13 month	Recover
Case9	Male	54	Deceased	2	Yes	Tac, Cor, MMF	TMP-SMX → Primaquine + Clindamycin	1.5 month	Die
Case10	Male	55	Deceased	1	No	Tac, Cor, MMF	TMP-SMX	29 month	Recover
Case11	Male	61	Living	1	Yes	Tac, Cor, MMF	TMP-SMX	5.7 month	Recover
Case12	Male	43	Living	1	Yes	Tac, Cor	TMP-SMX	6.1 month	Graft fail
Case 13	Female	25	Living	0	Yes	Tac, Cor, MMF	TMP-SMX	—	Recover
Case 14	Male	53	Deceased	2	No	Tac, Cor, MMF	TMP-SMX	2.8 month	Recover
Case 15	Male	52	Living	2	Yes	Tac, Cor, MMF	TMP-SMX	4.8 month	Recover
Case 16	Female	56	Deceased	2	Yes	Tac, Cor, MMF	TMP-SMX → Primaquine + Clidamycin	17 month	Recover
Case 17	Male	65	Deceased	0	No	Tac, Cor, MMF	TMP-SMX	—	Recover
Case 18	Male	40	Deceased	2	Yes	Tac, Cor, MMF	TMP-SMX	1.2 month	Recover

Abbreviations: PJP, *Pneumocystis jirovecii* pneumonia; CMV, cytomegalovirus; KT, kidney transplantation; MMF, mycophenolate mofetil; Cor, corticosteroid; Tac, tacrolimus; TMP-SMX, trimethoprim/sulfamethoxazole; Miz, Mizoribine; Cys, cyclosporine.

### Risk factors for PJP in the total population

Table 3 shows the risk factors for PJP. In the univariate analysis, more men than women developed PJP, although this was not statistically significant (60.6% *vs*. 83.3%, respectively, *P* = 0.052). Median age, duration of follow up, graft source (deceased *vs*. living donor), proportion of re-transplant patients, and smoking status did not differ significantly between those who had PJP and those who did not. The underlying cause of kidney disease, necessitating transplantation, varied. However, hypertension, diabetes mellitus (DM), and IgA nephropathy were the major causes in both groups (*P* = 0.102). More acute graft rejection after transplantation wwere observed in the PJP group compared with the PJP negative group (83.3% *vs*. 20.1%, respectively, *P* < 0.001). And CMV infection was more common in the PJP group than the PJP negative group (61.1% *vs*. 12.9%, respectively, *P* < 0.001). Acute rejection and CMV infection were checked until PJP pneumonia in PJP group. Among those patients in the PJP group, 50% used medication for DM, compared to 26.1% in the PJP negative group (*P* = 0.032). Lowest lymphocyte counts were lower in the PJP group than in the control group (0.3 × 10^3^/µL *vs*. 0.7 × 10^3^/µL, *P* = 0.007). In terms of immunosuppressive agents, patients received either tacrolimus- or cyclosporine-based regimens; the regimen used did not differ between the PJP group and the control group (*P* = 0.710). In the multivariate analysis, acute graft rejection [odds ratio (OR), 11.81; 95% confidence interval (CI), 3.06–45.57] and CMV infection (OR, 5.42; 95% CI, 1.69–17.93) were associated with PJP, whereas sex, age, body mass index (BMI), and graft source were not.

**Table 3:** Risk factors for PJP pneumonia in total population (univariate and multivariate analysis).

Variable	Univariate			Multivariate	
	PJP negative (n = 482)	PJP positive (n = 18)	*P*	*OR* (*95% CI*)	*P*
Men, sex, N (%)	292(60.6)	15 (83.3)	0.052	3.93 (1.00–15.47)	0.050
Age, years (median, range)	47.0 (18.0–71.0)	53.0 (25.0–65.0)	0.076	1.02 (0.96–1.08)	0.536
Follow up duration, month (median, range)	36.2 (18.7–54.6)	36.3 (18.4–54.5)	0.688		
BMI, kg/m2 (median, IQR)	22.2 (20.1–24.2)	21.1 (18.5–23.7)	0.200	0.94 (0.78–1.14)	0.525
KT type, living, N (%)	314 (65.1)	8 (44.4)	0.072	0.50(0.17–1.44)	0.198
Re-transplantation, N (%)	40 (8.3)	1 (5.6)	1.000		
Ever smoker, N (%)	133 (27.6)	6 (33.3)	0.594		
Primary underlying disease, N (%)			0.102		
Polycystic kidney	15 (3.1)	2 (11.1)			
HTN	194 (40.2)	4 (22.2)			
DM	81 (16.8)	4 (22.2)			
IgA nephropathy	60 (12.4)	3 (16.7)			
Autoimmune disease	6 (1.2)	1 (5.6)			
Chronic Glomerulonephritis	35 (7.3)	1 (5.6)			
Nephrotic syndrome	40 (8.3)	1 (5.6)			
Recurrent pyelonephritis/other	19 (3.9)	2 (11.1)			
Unknown	32 (6.6)	0 (0)			
Acute graft rejection, N (%)	97 (20.1)	15 (83.3)	<0.001	11.81 (3.06–45.57)	<0.001
CMV infection, N (%)	62 (12.9)	11 (61.1)	<0.001	5.42 (1.69–17.39)	0.005
BK virus infection, N (%)	88 (18.3)	7 (38.9)	0.058		
Using DM medication, N (%)	126 (26.1)	9 (50.0)	0.032	1.59 (0.51–5.02)	0.427
History of TB, N (%)	29 (6.0)	1 (5.6)	1.000		
Lowest lymphocyte, 10^3^/*μ* $\ell$ (median, IQR)	0.7 (0.4–1.2)	0.3 (0.1–0.6)	0.007	1.21 (0.83–1.70)	0.290
Immunosuppressive agent, N (%)			0.710		
Cyclosporine based regimen	59 (12.2)	1 (5.6)			
Tacrolimus based regimen	423 (87.8)	17 (94.4)			

Abbreviations: PJP, *Pneumocystis jirovecii* pneumonia; OR, odds ratio; CI, confidence interval; IQR, interquartile range; BMI, body mass index; KT, kidney transplantation; TB, tuberculosis; HTN, hypertension; DM, diabetes mellitus.

**Figure 1:**
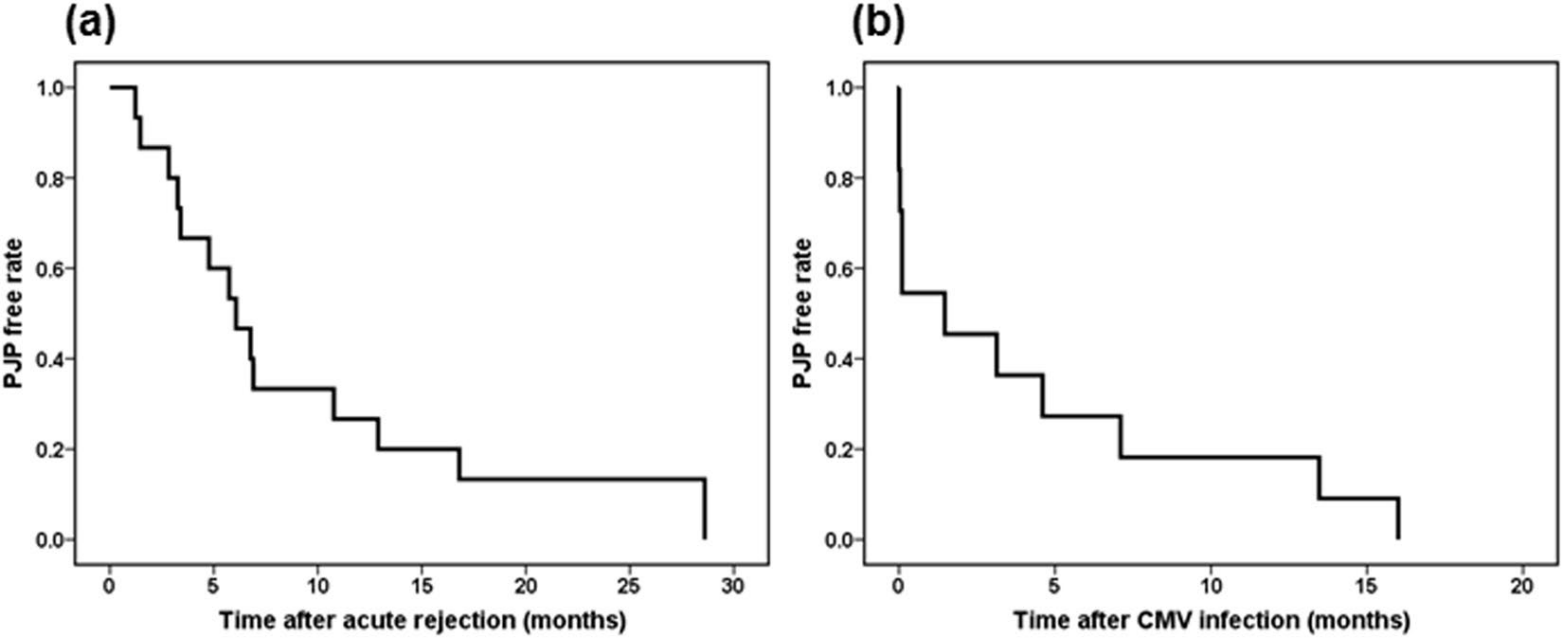
Interval time of PJP after acute graft rejection or CMV infection in PJP patients. (**a**) Acute rejection and PJP: Median month (IQR): 6.1 (3.3–12.9), (**b**) CMV infection and PJP: Median month (IQR): 1.5 (0.1–7.1). Note: PJP, *Pneumocystis jirovecii* pneumonia.

Acute graft rejection and CMV infection were important risk factors for PJP, as presented in Table 3. To understand the impact of acute graft rejection and CMV pneumonia on PJP, we investigated the timing of the onset of PJP after acute graft rejection or CMV infection. The median interval between acute graft rejection and PJP was 6.1 (IQR, 3.3–12.9) months, while the median interval between CMV infection and PJP was 1.5 (IQR, 0.1–7.1) months (Fig. 1a and b).

### Risk factors for PJP in patients experiencing acute graft rejection

As shown in Table 3, most cases of PJP (83.3%) occurred after acute graft rejection. Hence, we performed a subgroup analysis of the risk factors for PJP in patients who developed acute graft rejection (Table 4). Of the 500 kidney transplant patients, 112 experienced acute graft rejection. Of those, 15 were diagnosed with PJP. Sex, median age, BMI, graft source, smoking status, BK virus infection, use of DM medication, development of acute graft rejection within 1-year post-transplant, number of acute graft rejection episodes, and lowest lymphocyte count did not differ between patients who developed PJP and those who did not. All patients who developed acute graft rejection were managed with steroid pulse therapy. However, additional use of ATG for acute graft rejection treatment was more used in the PJP group (73.3% *vs*. 39.2%, *P* = 0.013). In addition, the proportion of patients with CMV infection was higher in the PJP group than in the PJP negative group (66.7% *vs*. 34%, respectively, *P* = 0.016). In the multivariate analysis, the independent risk factors for PJP in the acute graft rejection subgroup were identified as male sex (OR, 6.45; 95% CI, 1.24–33.73) and the use of ATG (OR, 5.25; 95% CI, 1.01–27.36).

**Table 4:** Risk factors for PJP in acute graft rejection patients (univariate and multivariate analysis).

Variable	Univariate			Multivariate	
	PJP negative (n = 97)	PJP positive (n = 15)	*P*	*OR* (*95% CI*)	*P*
Men, sex, N (%)	62 (63.9)	13 (86.7)	0.138	6.45 (1.24–33.73)	0.027
Age, years (median, IQR)	48.0 (38.5–55.0)	53.0 (43.0–55.0)	0.128	1.03 (0.97–1.10)	0.331
BMI, kg/m^2^ (median, IQR)	22.1 (20.2–24.3)	21.5 (18.6–23.7)	0.492	0.96 (0.77–1.18)	0.677
KT type, living, N (%)	60 (61.9)	7 (53.3)	0.264	0.59 (0.18–1.97)	0.394
Ever smoker, N (%)	27 (27.8)	5 (13.4)	0.760		
CMV infection, N (%)	33 (34.0)	10 (66.7)	0.016		
BK virus infection, N (%)	27 (27.8)	7 (46.7)	0.225		
Steroid pulse, N (%)	97 (100.0)	15 (100.0)	1.000		
Using DM medication, N (%)	34 (35.1)	7 (46.7)	0.385		
Using of ATG, N (%)	38 (39.2)	11 (73.3)	0.013	5.25 (1.01–27.36)	0.006
Acute graft rejection within 1 year after KT, N (%)	82 (84.5)	12 (80.0)	0.706		
Lowest lymphocyte, 10^3^/*μ* $\ell$ (median, IQR)	0.26 (0.1–0.6)	0.28 (0.1–0.4)	0.617		
Rejection number ≥2, N (%)	28 (28.9)	6 (40.0)	0.382		
Re-transplantation, N (%)	7 (7.2)	1 (6.7)	1.000		
History of TB, N (%)	29 (6.0)	1 (6.7)	1.000		
Immunosuppressive agent, N (%)			1.000		
Cyclosporine based regimen	7 (7.2)	1 (6.7)			
Tacrolimus based regimen	90 (92.8)	14 (93.3)			

Abbreviations: PJP, *Pneumocystis jirovecii* pneumonia; OR, Odds ratio; CI, confidence interval; IQR, interquartile range; BMI, body mass index; KT, kidney transplantation; DM, diabetes mellitus; ATG, anti-thymocyte globulin; TB, tuberculosis.

## Discussion

In our study, of 500 kidney transplant patients, 18 developed PJP. Acute graft rejection and CMV infection were identified as risk factors for PJP. Within the subgroup of patients who experienced acute graft rejection, male sex and the use of ATG were risk factors for PJP.

Our data showed that more patients in the PJP group than in the PJP negative group had CMV infection. This result is in agreement with that of previous studies^17, 21^. CMV is known to modify host immune responses by various mechanisms, suppressing helper T-cell and antigen presenting cell functions^22^. Therefore, CMV infection is both a marker of an immunocompromised state and has, itself, an immunosuppressive effect^23^. Therefore, when one opportunistic infection is diagnosed in transplant patients, physicians should consider that patients may at the same time or in the following weeks/months present with another opportunistic infection.

Several articles have reported that the development of acute graft rejection is a risk factor for PJP^10, 13, 15, 17^. Our data were similar. When acute graft rejection occurs, patients are treated with steroid pulse therapy or other immunosuppressive agents. These results support the hypothesis that severe immunosuppression may increase the occurrence of PJP. Similarly, low lymphocyte counts have been reported to be associated with the development of PJP^12^. Our data showed that the median lowest lymphocyte count was relatively low in the PJP group. Although this result was not evident in multivariate analysis, the univariate analysis still suggests the importance of lymphocytes in the development of PJP infection.

Previous studies have reported other risk factors for PJP in patients who have undergone kidney transplantation^3, 15, 24^. Lufft *et al*. first reported that different immunosuppressive regimens could affect the occurrence of PJP in renal transplant recipients^24^. In their report, tacrolimus-based regimens seemed more likely to trigger PJP, but this was not confirmed. MMF was suggested for use as an immunosuppressive agent having anti-PJP effects^25, 26^. In our study, there was no difference in the occurrence of PJP between the groups of patients receiving tacrolimus- or cyclosporine-based regimens. We could not analyze the effect of MMF on PJP because kidney transplant recipients at our center almost used MMF. Further research on the effect of immunosuppressive regimens on development of PJP is needed.

We performed further evaluation of patients (n = 112) who experienced acute graft rejection. Arend *et al*. reported that the incidence of PJP among patients treated for 0, 1, 2, or ≥3 rejection episodes increased with the increasing number of rejection episodes. The proportion of patients requiring additional ATG for an episode of rejection was higher in the PJP positive group than in the control group^27^. In our study, there was no association between the number of acute graft rejection episodes and PJP. Only additional use of ATG for treatment of acute graft rejection was associated with the development of PJP in the acute graft rejection subgroup. PJP occurred in four patients whilst taking PJP prophylaxis, three of whom had used additional ATG treatment prior to developing PJP. These results suggest that immunosuppressive status is more important than other factors in the development of PJP.

All guidelines recommend PJP prophylaxis for a certain period after kidney transplantation, although no universal consensus exists on the optimal duration of prophylaxis^8–10^. Our data indicated that acute graft rejection, CMV infection, and ATG use in the acute graft rejection subgroup could be the risk factors for PJP. Thus, further PJP prophylaxis is suggested in patients who have these risk factors, based on our data. In terms of the duration of prophylaxis, most cases of PJP occurring after acute graft rejection occurred within 12 months, and most cases of PJP occurring after an episode of CMV infection occurred within 6 months. Hence, we suggest a 6–12 month course of prophylaxis following either an episode of acute graft rejection or CMV infection. Although some complications have been reported with the use of TMP/SMX for PJP prophylaxis, major complications are relatively rare^28^; therefore, the benefits of PJP prophylaxis outweigh the risks.

There are several limitations to our study. First, we used the results of PCR as well as direct immunofluorescence to diagnose PJP. Thus, our data might include patients with false positive results. In general, confirmation of PJP requires special microbiological stains of sputum or bronchoalveolar lavage (BAL) specimens^29^. However, the proportion of PJP cases in HIV-uninfected patients in which is confirmed as organism in respiratory specimens is relatively low (compared with HIV-infected patients) because of the low burden of organism^30–32^. *Pneumocystis jirovecii* PCR assays are sensitive and increase the diagnostic yield in HIV-uninfected immunocompromised patients^33–35^. We used chest CT findings at initial diagnosis of PJP to exclude patients with false positive *Pneumocystis jirovecii* PCR results. Second, the number of PJP patients in our study was relatively small. However, unlike other studies, our study collected data on a large number of consecutive kidney transplant patients, instead of selecting particular patients; this point can be its strength. Third, patients with CMV infection and patients who used ATG overlapped in the acute graft rejection subgroup. Thus, we could not analyze the independent effect of CMV infection or ATG use on PJP. Fourth, this study was retrospective, thus, we could not systemically analyze multiple laboratory test results. There is a need for further, prospective studies to assess the risk factors for PJP.

## Conclusion

Our data suggest that acute graft rejection and CMV infection may be risk factors for PJP in kidney transplant patients. In patients who develop acute graft rejection, the use of ATG may increase the risk of PJP. We suggest providing further PJP prophylaxis for kidney transplant patients who develop CMV infection or acute graft rejection, especially for those treated with ATG.
